# Psychotherapeutic Group Intervention for Traumatized Male Refugees Using Imaginative Stabilization Techniques—A Pilot Study in a German Reception Center

**DOI:** 10.3389/fpsyt.2018.00533

**Published:** 2018-10-29

**Authors:** Catharina Zehetmair, Claudia Kaufmann, Inga Tegeler, David Kindermann, Florian Junne, Stephan Zipfel, Sabine C. Herpertz, Wolfgang Herzog, Christoph Nikendei

**Affiliations:** ^1^Department of General Internal Medicine and Psychosomatics, Center for Psychosocial Medicine, University Hospital of Heidelberg, Heidelberg, Germany; ^2^Department of General Psychiatry, Center for Psychosocial Medicine, University Hospital of Heidelberg, Heidelberg, Germany; ^3^Department of Psychosomatic Medicine and Psychotherapy, Medical University Hospital, Tübingen, Germany

**Keywords:** imaginative stabilization techniques, refugees, post-traumatic stress disorder, group therapy session, reception center

## Abstract

**Background:** Due to persecution, human rights violations and armed conflicts, the prevalence of post-traumatic stress disorder (PTSD) is high in refugee populations. Previous studies indicate that trauma-focused treatments are highly effective in treating PTSD in refugees. However, these approaches rely on the stability of the therapeutic setting, treatment continuity, and safe housing. Although early treatment of PTSD is recommended, these requirements are not met in reception centers. Therefore, we conducted a pilot study to examine the effect of imaginative stabilization techniques derived from psychodynamic psychotraumatology therapy for the early stabilization of traumatized refugees in a reception center.

**Methods:** From May 2017 to April 2018, 86 imaginative stabilization group therapy sessions have taken place. A sample of 43 out of 46 traumatized refugees completed self-report questionnaires assessing PTSD, depression, and anxiety symptoms prior to attending open imaginative stabilization group therapy sessions. Furthermore, participants filled in self-report questionnaires on distress and emotional state (valence/arousal/dominance) before and after each session. After having participated in four consecutive sessions, a sub-group of 17 participants completed a follow-up assessment of PTSD, depression, and anxiety symptoms. Follow-up interviews were conducted with 25 participants 2 weeks after their last session attendance to explore self-practice habits post intervention.

**Results:** The pre-post-intervention comparison of scores indicated a significant reduction of distress (*z* = −3.35, *p* < 0.001, *r* = −0.51) and an improvement of affective reports for valence (*z* = −4.79, *p* < 0.001, *r* = −0.82) and dominance (*z* = −3.89, *p* < 0.001, *r* = −0.59), whereas arousal scores were not affected. We found a significant reduction of anxiety symptoms (*z* = −2.04, *p* < 0.05, *r* = −0.49), whereas PTSD and depression scores remained unchanged. Follow-up interviews revealed that 80% of the participants continued to practice the imaginative stabilization techniques after redistribution to other accommodation.

**Conclusion:** The results indicate that imaginative stabilization techniques are a promising and feasible approach to treat refugees in unstable reception center settings. In regular imaginative stabilization group therapy sessions, we were able to reduce the participants' distress and anxiety symptoms while strengthening their internal resources and increasing their emotional stability.

## Introduction

As a result of persecution, armed conflicts, deficient health care, and human rights violations, more than 65.5 million people worldwide were forcibly displaced from their homes by the end of 2016. Approximately one third of these refugees sought safety in Europe, risking their lives during a perilous journey (1). Between 2016 and 2017, around 920,000 first-time asylum applicants were registered in Germany (2). Due to pre-, peri-, and post-migratory distress factors, fleeing people show a high risk to develop mental health problems. In recent literature, up to 40% of refugees are reported to be affected by post-traumatic stress disorders (PTSD), depression, and anxiety disorders (3–8). Observed prevalence rates of mental health problems in refugees are inconsistent due to the heterogeneity of the groups and to the diversity of applied diagnostic instruments (9, 10). Regardless of the exact numbers, current findings underline the urgent need for culturally sensitive interventions shortly after the refugees' arrival in the respective host countries to enable sufficient treatment of mental health problems and prevent further exacerbation and chronification.

Even though PTSD prevalence rates in refugees are high, research on the effectiveness and efficacy of different therapeutic approaches for refugees is still lacking (11–14). To date, narrative exposure therapy (NET) is the most closely investigated approach for the treatment of PTSD with well-documented evidence, including meta-analytical reviews, indicating the effective reduction of PTSD symptoms (15–23). Further, studies investigating trauma-focused behavioral therapy (24–27) and eye movement desensitization and reprocessing [EMDR; (28, 29)] also show results that point to a significant decrease of PTSD symptoms.

All the interventions described above directly target traumatic experiences in order to achieve symptom control and psychological stabilization. This requires a safe, stable, and continuous psychotherapeutic setting. However, upon arrival in their countries of destination, refugees are often initially sheltered in reception centers where psychosocial and psychiatric evaluation and mental health care provision is sparse, although urgently needed (5, 7, 14, 30, 31). The implementation of interventions for traumatized refugees is further limited through the redistribution to collective or municipal accommodations. Especially the setting provided by accommodation in reception centers is not stable enough for trauma confrontation treatment. This in turn underpins the need for specific therapeutic approaches that might prove to be adaptive for this specific setting.

Imaginative stabilization techniques derived from psychodynamic trauma therapy (32) might represent one possible strategy to support traumatized refugees. This approach aims to develop, activate, and strengthen individual skills, resources, and coping strategies, as well as to improve self-care and -calming skills by the means of imagining self-assuring and -activating inner pictures (14, 32). As cornerstones of psychodynamic imaginative trauma therapy [PITT; (33)], imaginative stabilization techniques have been shown to significantly reduce trauma symptoms in psychiatric inpatients (34, 35). Studies with refugee samples apply imaginative stabilization techniques either as one core element within a multimodal stabilization approach (36), or as a part of different symptom management techniques before trauma confrontation (37). These studies point out that imaginative stabilization techniques are applicable for refugee populations. However, so far, the specific effect of imaginative stabilization techniques on PTSD symptom reduction has not been addressed. Further research on stabilization interventions among traumatized refugees has revealed inconsistent results without specifying what exactly is meant by the term “stabilization” (29).

Therefore, this pilot study aims to investigate the feasibility and effectiveness of imaginative stabilization techniques (32) for traumatized, English-speaking, male refugees sheltered in a German reception center in a group therapy setting using a quantitative pre-post design. As primary outcome measures we hypothesized that the imaginative stabilization techniques would (1) positively impact the refugees' affective state and (2) decrease their perceived distress. Our secondary outcome measure was the assumption that the intervention would lead to symptom reduction, reflected in (3) a decrease in PTSD symptoms as well as (4) an improvement of psychiatric comorbidities in terms of depression and anxiety symptoms. Finally, (5) we assumed that the refugees would practice the imaginative stabilization techniques on their own after their last imaginative stabilization group therapy session attendance.

## Materials and methods

### Participants

Over a period of one year (from the end of May 2017 to the end of April 2018), refugees that consulted the psychosocial outpatient clinic (30, 31) in the reception center “Patrick Henry Village” (PHV) in Heidelberg Kirchheim, Germany, and met the inclusion criteria were referred to our imaginative stabilization group therapy sessions. All participating refugees had either applied for asylum in Germany before the intervention or were in the middle of the application process during the intervention. Study inclusion criteria were: diagnosed PTSD by experienced psychotherapists and psychiatrists working in the psychosocial outpatient clinic, male gender, and the ability to speak and understand the English language. Exclusion criteria were addiction, current psychosis, traumatic brain injury, and age below 18 years.

### Study design and ethical approval

We investigated the feasibility and effectiveness of imaginative stabilization techniques (32) in refugees suffering from PTSD and sheltered in a reception center using a prospective, longitudinal design. As the participants' imminent redistribution to other accommodations caused a high degree of fluctuation, we chose an open group setting. We decided against a mixed-gender concept to ensure a comfortable, safe, and trusting environment with minimal triggering potential. Due to the fact that a higher portion of male patients sought help at the reception center's psychosocial outpatient clinic (30) and the center's existing psychosocial offers (e.g., shelters for women and children offered by charitable organizations) catered to the needs of burdened women, we decided on a group therapy setting exclusively for male refugees. The following assessment points were defined:

- *Baseline measurement of the severity of symptoms related to mental distress:* Prior to the individuals' first imaginative stabilization group therapy session, we used a questionnaire to assess symptoms of PTSD, depression, and anxiety disorders.
- *Pre-post imaginative stabilization group therapy session assessment:* Additionally, pre-post effects of imaginative stabilization techniques on the refugees' affective state and their perceived distress were examined.
- *Follow-up symptom severity measure of mental distress for subgroup:* Furthermore, we used the same questionnaire as we had used for the baseline measurement to assess symptom reduction in PTSD, depression, and anxiety disorders in a subgroup of participants after they had attended the imaginative stabilization group therapy sessions for the fourth time.
- *Follow-up interviews:* Finally, follow-up interviews were carried out with participants via phone 2 weeks after their last imaginative stabilization group therapy session attendance, in order to assess why they had stopped coming to the group and whether they had continued to practice the techniques individually.

The study was approved by the ethics committee of the University of Heidelberg (S-640/2016) and all participants gave their written informed consent in accordance with the Declaration of Helsinki.

### Imaginative stabilization group therapy sessions

The imaginative stabilization group therapy sessions took place twice a week and were carried out by two psychotherapists: a post-doctoral scientist and licensed psychodynamic psychotherapist highly experienced in trauma therapy, and a Heidelberg University Ph.D. candidate undergoing post-graduate training in behavioral therapy. Before the participants' first session, the therapists individually met new group members to inform them about the structure and the content of the group setting. Participants who had already attended the imaginative stabilization group therapy sessions at least once, filled out the questionnaire at the beginning of the session. We started the imaginative stabilization group therapy sessions by asking the participants how they were feeling and giving them the possibility to talk about their individual difficulties or ask questions. Next, we began with the three imaginative stabilization techniques. The first technique consisted of mindful breathing to enable the participants to relax, focus on the present moment, and enter a state of self-engagement. The second technique, the Body Scan which was originally developed by Kabat-Zinn (38) and described by Reddemann (32), aims to teach the participants self-perception and enable them to experience their bodies by mindfully focusing on different parts of their body. For the third technique we used one of two guided imagery techniques: either the “inner safe place” or the “tree exercise” (32). The technique “inner safe place” encourages individuals to imagine themselves at a real or made-up place where they feel comfortable and safe. During the “tree exercise,” individuals imagine a tree as a symbol of strength and nutrition with the goal to evoke a feeling of inner stability, comfort, and energy. Both exercises focus on different sensory perceptions aimed at deepening the imagination and strengthening feelings of safety and comfort. After having completed all three techniques, participants had the possibility to share their experiences in a short discussion round. At the end of each imaginative stabilization group therapy session the participants filled out questionnaires.

### Measures

#### Baseline measurement of the severity of symptoms related to mental distress

Prior to the first imaginative stabilization group therapy session, a baseline measurement (T1) including the Primary Care PTSD Screen for DSM-5 [PC-PTSD-5; (39)], the two-item Patient Health Questionnaire [PHQ-2; (40)], and the short version of the General Anxiety Disorder questionnaire [GAD-2; (41)] were used to assess mental distress of all participants.

We assessed the individuals' PTSD symptoms via the PC-PTSD-5 (39). Derived from DSM-5 criteria, the questionnaire begins with a list of trauma events which we adapted to include the trauma most frequently experienced by refugees (14). Items are assessed by a dichotomous (yes/no) response format instead of a Likert scale to facilitate administration and scoring. Individuals who do not report trauma exposure do not answer subsequent questions on PTSD symptoms. If participants confirmed at least one trauma event, they were asked five binary questions to assess their perception of these experiences: (1) Were they re-experiencing the trauma? (2) Were they avoiding situations related to the trauma? (3) Were they experiencing recurrent thoughts about the trauma? (4) Were they suffering emotional numbness? (5) Were they feeling guilty of the experiences? (39). The PC-PTSD-5 has possible scores between 0 and 5 and a cut off score at 3. It shows good sensitivity (0.93) and acceptable specificity (0.85) and was well tolerated by patients (42). The average Kuder-Richardson formula 20 reliability coefficient was 0.52.

In the PHQ-2 (40) participants are asked two questions on anhedonia and depressed mood. The possible answers are set on a scale between 0 (not at all) and 3 (nearly every day). The overall score of the PHQ-2 ranges between 0 and 6 and has a good construct validity (r from 0.67 to 0.87) and a good internal consistency [α = 0.83; (43)]. The cut-off score set at ≥3 shows a sensitivity of 0.61–0.87 and a specificity of 0.86–0.92 for major depression in primary care and medical outpatients (40, 43, 44), and a sensitivity of 0.79 and a specificity of 0.86 for any other depressive disorder (43).

The GAD-2 (41) is recommended by the National Institute for Health and Care Excellence Guidelines (45) for the detection of anxiety disorders. It consists of two items: (1) “Feeling nervous, anxious or on edge” and (2) “Not being able to stop or control worrying” with response possibilities from 0 (not at all) to 3 (nearly every day). The overall score ranges between 0 and 6. With the cut-of score set at ≥3 the GAD-2 shows a sensitivity of 0.89 for generalized anxiety disorder and a specificity of 0.83 for generalized anxiety disorder (41, 46). Internal consistency reliability is acceptable [α = 0.83; (47)].

#### Pre-post imaginative stabilization group therapy session assessment

As primary outcome measure we administered the Self-Assessment Manikin scale [SAM; (48)] and the distress thermometer of the Refugee Health Screener-15 [RHS-15 distress thermometer; (49)] before (pre) and after (post) each imaginative stabilization group therapy session.

The SAM (48) is a nonverbal, cross cultural (50) rating scale. Patients are asked to select one out of five manikin pictures to describe their present affective state concerning the three major affective dimensions of valence (sad—happy), arousal (exited—calm), and dominance (weak—strong). The dimension of valence ranging from 1 (sad) to 5 (happy) illustrates the participants' positive or negative feelings; the dimension of arousal ranging from 1 (excited) to 5 (calm) describes their perceived vigilance and excitement; the dimension of dominance ranging from 1 (weak) to 5 (strong) represents their feeling of being in control of the current situation (51). The SAM scales are widely used in different situations and for diverse groups of patients, i.e. for traumatized patients as well as for refugees (51–54).

The RHS-15 (49) was developed especially for refugees and asylum seekers. It contains 14 symptom items and a distress thermometer which rates distress on a visual analogous scale ranging from 0 (“Things are good”) to 10 (“I feel as bad as I ever had”). With a cut-off score set at ≥5, sensitivity (0.81–0.95) and specificity (0.86–0.89) of the RHS-15 are good (49).

#### Follow-up symptom severity measure of mental distress for subgroup

When individuals had participated in four imaginative stabilization group therapy sessions, they were asked to fill out the PC-PTSD-5 (39), PHQ-2 (40), and GAD-2 (41) again, in order to compare the rate of their mental distress after four sessions (T2) to their answers prior to the first session (T1) as secondary outcome measures.

#### Follow-up interviews

We contacted all participants via phone two weeks after their last imaginative stabilization group therapy session attendance. The phone calls were digitally recorded in order to ensure a reliable analysis. The three key questions of these interviews were developed according to the guidelines of the COREQ-Checklist (55) and designed as semi-structured interviews according to the methodological aspects of Helfferich (56). With our first question, we aimed to discover the reasons why the participants had stopped coming to the imaginative stabilization group therapy sessions. Secondly, we asked the participants whether they were practicing the imaginative stabilization techniques on their own. The third question concerned frequency, time and preferred place of self-practice.

### Data analysis

All statistical analyses were carried out by using the Statistical Package for the Social Sciences (SPSS) program version 24 (57). Demographic variables and baseline characteristics were analyzed using descriptive statistics (frequencies, means, and standard deviations). Because the data distributions tended to be slightly skewed, we used Wilcoxon signed-rank tests. We compared the scores of the SAM and the RHS-15 distress thermometer before an individual's first (pre) and after an individual's last (post) imaginative stabilization group therapy session attendance (primary outcome measures). The baseline scores for PTSD, depression, and anxiety disorders (T1) were compared with the scores after the fourth imaginative stabilization group therapy session (T2) for the sub-sample (secondary outcome measure). Effect sizes were calculated using Pearson's correlation coefficient *r*. The internal consistency reliability of PC-PTSD-5 was determined using Kuder-Richardson formula (KR20). The recorded follow-up interviews were transcribed and analyzed descriptively by using frequency analyses in MAXQDA (58) according to the qualitative content analyses of Mayring (59).

## Results

### Characteristics

During the study period a total of 86 imaginative stabilization group therapy sessions took place and *N* = 46 participants visited the sessions at least once. On average, the participants attended 5.43 sessions (SD = 7.34, range 1–40). Eight participants (17.4%) attended 10 or more sessions, whereas 26 participants (56.5%) took part in the imaginative stabilization group therapy sessions less than four times. Regarding the attendance of each session, we calculated that on average three participants (*M* = 2.92, *SD* = 1.67, range 0–10) took part in a group session. For the statistical analysis that is described below, two participants had to be excluded due to their high level of mental distress and danger of further exacerbation when confronted with the questionnaires. A further participant was excluded from the statistical analysis because he reported psychotic symptoms during the time of his imaginative stabilization group therapy session attendance. All in all, there was a sub-sample of *n* = 17 participants who filled out the questionnaires regarding PTDS, depression, and anxiety disorders after the fourth session, thus enabling us to assess their follow-up symptom severity. Table 1 depicts sample characteristics for all participants as well as for the sub-sample of participants (*n* = 17) who joined at least four imaginative stabilization group therapy sessions.

**Table 1:** Sociodemographic characteristics at baseline for all participants (*N* = 46) and the sub-sample (*n* = 17).

	**At baseline^a^**			**Sub-Sample^b^**		
	***n***	***%***		***n***	***%***	
**REGION OF ORIGIN**						
Sub-Saharan Africa	33	71.7		14	82.4	
Middle East	5	10.9		2	11.8	
South Asia	6	13.0		1	5.9	
North-Africa	2	4.3		
**RELIGION**						
Christian	27	58.7		12	70.6	
Muslim	15	32.6		4	23.5	
Hindu	2	4.3		1	5.9	
Atheist	2	4.3		
**MEDICATION^C^**						
None	12	26.1		7	36.8	
Antidepressants	32	69.6		9	52.9	
Neuroleptics	6	13.0		1	5.9	
No information	1	2.2		
	***M***	***SD***	**Range**	***M***	***SD***	**Range**
Age	27.50	7.47	18–45	25.24	4.87	18–32
Stay in PHV (weeks)	9.46	8.31	1–32	7.35	5.00	2–16
Session attendance	5.43	7.34	1–40	10.88	9.70	4–40

a *N = 46*;

b *n = 17 participants who attended four or more imaginative stabilization group therapy sessions; PHV,reception center for refugees “Patrick-Henry Village” in Heidelberg Kirchheim, Germany; Session attendance,participation in imaginative stabilization group therapy sessions*;

c *Multiple answers were possible*.

### Baseline measurement of the severity of symptoms related to mental distress

The baseline scores for PTSD, depression, and anxiety disorders are presented in Table 2. On average, the participants reported four trauma experiences (*N* = 43, *M* = 4.12, *SD* = 1.65, range = 1–8). The most frequent traumatic events were seeing someone being killed or seriously injured (*n* = 33, 76.7%), experiencing torture (*n* = 29, 67.4%), being physically or sexually assaulted or abused (*n* = 27, 62.8%), experiencing a war (*n* = 19, 44.2%), being imprisoned (*n* = 25, 58.1%), and losing a loved one through homicide or suicide (*n* = 17, 39.5%). Beyond that, 34 participants (79.1%) fulfilled the criteria of a major depression and 38 participants (88.4%) displayed symptoms of anxiety disorders.

**Table 2:** Baseline measurement of all participants and T1-T2 comparison of sub-sample for PTSD, depression, and anxiety disorders.

	**All participants^a^**		**Sub-Sample^b^**			
			**T1**		**T2**				**Pearson's**
	***M***	***SD***	***M***	***SD***	***M***	***SD***	***z***	***p^c^***	***r***
PC-PTSD-5	3.78	1.20	3.65	1.22	3.10	0.97	−1.93	0.06	−0.47
PHQ-2	3.79	1.55	4.06	1.82	3.35	1.54	−1.34	0.20	−0.32
GAD-2	4.28	1.52	4.47	1.59	3.82	1.59	−2.04	< 0.05	−0.49

a *N = 43*;

b *n = 17 participants who attended four or more imaginative stabilization group therapy sessions*;

c *p,exact significance (two-tailed); PC-PTSD-5, Primary Care Posttraumatic Stress Disorder Screen for Diagnostic and Statistical Manual of Mental Disorders 5; PHQ-2, two-item Patient Health Questionnaire; GAD-2, short version of the General Anxiety Disoder-7; T1, rating prior to the first imaginative stabilization group therapy session attendance; T2, rating after the fourth imaginative stabilization group therapy session attendance*.

### Pre-post imaginative stabilization group therapy session assessment

Table 3 displays the mean scores for the pre- to post comparison in current affective state (SAM) and perceived distress (RHS-15 distress thermometer). Comparing scores of the SAM item “valence” prior to the participants' first (*M* = 4.30, *SD* = 1.26) and after their last imaginative stabilization group therapy session (*M* = 2.88, *SD* = 1.14), the pre-post difference turned out to be significant (*z* = −4.79, *p* < 0.001. *r* = −0.82). There was no effect on the SAM item “arousal” scores (*z* = 0.21, *p* = 0.83, *r* = −0.03). The third SAM item “dominance” showed a significant difference (*z* = −3.89, *p* < 0.001, *r* = −0.59). Scores of the RHS-15 distress thermometer showed a significant decrease of perceived distress (*z* = −3.35, *p* < 0.001, *r* = −0.51).

**Table 3:** Pre-post imaginative stabilization group therapy session assessment of perceived distress and current emotional state.

	**Pre**		**Post**				**Pearson's**
	***M***	***SD***	***M***	**SD**	***z***	***p^a^***	***r***
SAM
Valence	4.30	1.26	2.88	1.14	−4.79	< 0.001	−0.82
Arousal	3.00	1.59	3.02	1.12	−0.21	0.85	−0.03
Dominance	2.12	1.28	3.12	1.12	−3.89	< 0.001	−0.59
RHS-15 distress thermometer	6.57	2.80	5.14	2.67	−3.35	< 0.001	−0.51

*N = 43*;

a *p,exact significance (two-tailed); SAM, Self-Assessment Manikin; RHS-15,Refugee Mental Health Screener-15; Pre, rating prior to the first imaginative stabilization group therapy session attendance; Post,rating after the participants' last imaginative stabilization group therapy session attendance*.

### Follow-up symptom severity measure of mental distress for sub-group

Table 2 depicts the mean values for the T1-T2 comparisons of PTSD, depression, and anxiety disorders, as well as calculated changes in symptom severity. The Wilcoxon signed-rank test did not show a statistically significant change in PTSD scores between the first and after the fourth imaginative stabilization group therapy session (*z* = −1.93, *p* = 0.06, *r* = −0.47). The calculated comparison between T1 and T2 depression scores did not reveal a significant difference of depression scores (*z* = −1.34, *p* = 0.18, *r* = −0.32), but showed a significant reduction for anxiety scores (*z* = −2.04, *p* < 0.05, *r* = −0.49).

### Follow-up interviews

In total, we made three attempts to reach each participant by telephone. *n* = 10 (24%) of the participants did not have a cellphone. We were able to conduct follow-up interviews with 25 (58%) out of 43 participants. Most participants (*n* = 23, 92%) informed us that they had not been able to attend the imaginative stabilization group therapy sessions anymore because of having been transferred to collective or municipal accommodations. *n* = 20 (80%) of the participants reported having continued to practice the imaginative stabilization techniques on their own, *n* = 4 (20%) participants had not practiced at all and *n* = 1 participant did not respond to any of the interview questions. Table 4 lists the answers of all participants we interviewed. The majority of the participants practiced the mindful breathing exercise (95%). The Body Scan (55%) and the guided imagery techniques (45%) were practiced approximately equally often. Participants preferred practicing the imaginative stabilization techniques either before sleeping (45%) or regardless of the time of day whenever symptoms of distress occurred (30%). Most of the participants chose to do the techniques in their rooms (45%) or outside (10%).

**Table 4:** Report of the follow-up interviews for all participants who were contacted via phone (*N* = 25) and further details about self-practice among participants practicing the techniques (*n* = 20).

	**All participants^a^**	
	***n***	***%***
Amount of practice		
Several times a day	4	16
Once a day	3	12
Two or three times a week	5	20
Sometimes	8	32
Never	4	16
No information	1	4
	**Participants practicing the techniques**^b^	
Type of technique^c^		
Breathing	19	95
Body scan	11	55
Guided imagery technique	9	45
Daytime^c^		
Morning	4	20
Afternoon	1	5
Evening	3	15
Night	9	45
If symptoms get worse	6	30
No information	3	15
Location		
Room	9	45
Outside	2	10
No information	9	45

a *N = 25*;

b *n = 20*;

c *Multiple answers were possible*.

## Discussion

In this pilot study, we aimed to examine the effectiveness of imaginative stabilization techniques among refugees sheltered in a reception center using a pre-post design. Regarding primary outcome measures, our results showed that imaginative stabilization techniques were effective in increasing the perception of positive feelings and being in control, as well as reducing distress. The intervention did not affect arousal. In addition, results of secondary outcome measures showed that anxiety symptoms were reduced significantly by the techniques, whereas PTSD symptoms and depression scores remained unchanged. The follow-up interviews indicated that a majority of the participants continued to practice imaginative stabilization techniques even when they were no longer able to attend the imaginative stabilization group therapy sessions anymore. The results demonstrate that group therapy which is focused on practicing imaginative stabilization techniques with refugees sheltered in a reception center is a feasible intervention which can improve the refugees' mental health despite unstable conditions.

Results of the Self-Assessment Manikin scale depicted that participation in the imaginative stabilization group therapy sessions increased positive feelings and the feeling of being in control while not having any impact on the state of arousal. Positive feelings are a source of resilience, facilitating positive reappraisal, broadening cognitive flexibility, and positive responsiveness to stressors, whereas negative feelings narrow an individual's perception and thinking (60, 61). The link between imaginative stabilization techniques and positive feelings is congruent with the aim of imaginative stabilization techniques to increase self-efficacy and to strengthen individual resources. Some studies argue that the improvement of emotional states may prevent a relapse of PTSD (62). In addition, higher levels of positive emotions are negatively correlated with depressive symptoms (63). The lack of change in depression scores in our study might be ascribed to the low intervention dose.

After having participated in our imaginative stabilization group therapy sessions, we found participants' ego functions to have increased, which was reflected in an improved sense of self-efficacy. Loss of control and overwhelming emotions are common and well documented characteristics of traumatic experience. Furthermore, post-migratory stressors, such as uncertain future perspectives, resident status, and an altered, more negative world view maintain feelings of loss of control. It has been shown that these factors mediate and maintain PTSD and depression in refugees (64). Thus, it is especially noteworthy that the imaginative stabilization techniques could reinstall or at least improve the participants' perceived self-efficacy. It has also been shown that the perception of being in control can serve as a resilience factor in response to traumatic situations (61, 62, 65).

We were able to demonstrate that imaginative stabilization group therapy sessions reduced the refugees' distress level which is in line with findings of Kruse et al. (36). As Laban et al. (60) pointed out, techniques focusing on the connection between body and mind, the awareness of the present moment, and a non-judgmental perception of inner experiences increase participants' ability to cope with stress. Furthermore, self-efficacy is a protective factor because it enables management of stressful situations (61). Also, the psychosocial support the participants received by the therapists may have facilitated dealing with stress and daily hassles. Considering that many studies report refugees suffering from distress due to post-migratory factors and prolonged insecurity (66), the imaginative stabilization techniques can be an effective approach supporting newly arrived refugees in uncertain conditions by strengthening internal psychological resources.

Due to the high impact of imaginative stabilization techniques on affective state and perceived distress one might speculate that they should have had a similar effect on the PTSD symptom severity as well. However, we were not able to detect significant changes in PTSD symptomatology. Through imaginative stabilization techniques, participants learn how to self soothe, be aware of themselves in the present moment and how to deal with overwhelming emotions. As regular practice increases the beneficial impact of these techniques, our findings of persisting trauma symptoms may be explained through the dose of our intervention which was dependent on external circumstances in this study. Therefore, it is not surprising that current arousal levels assessed in the study did not differ significantly after the fourth imaginative stabilization group therapy session. In addition, the period of time until refugees are granted asylum is a phase of de-stabilization and re-traumatization because of the prolonged condition of insecurity, sense of powerlessness and preoccupation with past traumatic events, i.e., in the personal interviews which are part of the asylum procedure (67). Studies examining the effect of post-migratory distress factors on refugees' mental health conditions have previously outlined the importance of refugee support offers in the early stages of their asylum procedure (67). It is also important to note that the refugees who participated in our study had only recently arrived in Germany and were still waiting for an answer concerning their request for asylum. Hereby, efforts toward the reduction of traumatic symptoms may be understood as decreasing their chance of being granted asylum in Germany (36, 68, 69).

With regard to depression, imaginative stabilization techniques did not have a significant effect. These techniques have been developed specifically for the treatment of PTSD. However, some studies have shown that imaginative stabilization techniques may also have effects on depression in the context of psychodynamic imaginative trauma therapy (35). As traumatic stress can lead to symptoms of depression, it is not surprising that psychodynamic imaginative trauma therapy may also reduce the levels of depression (6). One could argue that the imaginative stabilization techniques help individuals to reduce depressive symptoms by encouraging them and increasing their self-efficacy. Nevertheless, negative self-perception due to loss of status and social networks, negative thoughts about life due to separation from family, loss or death of relatives and friends, as well as an uncertain future may provoke or aggravate depression (17, 67, 70, 71). Furthermore, current research emphasizes that traumatic loss and grief are core elements for the development of comorbid depression in traumatized refugees (72, 73).

Practicing imaginative stabilization techniques led to a significant reduction of anxiety symptoms. Our results suggest that increased self-efficacy and activation of resources may in turn decrease anxiety symptoms, even though PTSD symptoms and arousal remain unchanged. Further studies indicate that interventions which focus on mindfulness decrease anxiety symptoms by bringing the participants' awareness to the current moment and connecting to the here and now (62).

In the follow-up interviews we learnt that the imaginative stabilization techniques were well received by the participants. Most of them continued practicing the techniques when they were not able to participate in the imaginative stabilization group therapy sessions anymore. It is interesting to note that the majority of participants either used the techniques as a routine in their daily lives or as an intervention to control certain symptoms. The fact that the participants' preferences for specific techniques differed in this sample reflects the diversity of the refugee population. To cater for this diverse group of traumatized human beings, individuals should be encouraged to practice the technique(s) perceived as most helpful by them.

### Limitations

This study has several limitations. First, using a longitudinal approach with a small sample size means that results cannot be generalized without further research. Working with refugees in reception centers poses ethical barriers as well as difficulties related to the fact that most refugees are redistributed to other accommodation after a short period of time and without forewarning. Therefore, we planned our project as a pilot study to create a basis for further research in which the effectiveness of our intervention can be assessed using a randomized, controlled trial. All participants referred to the imaginative stabilization group therapy sessions participated in the study and most of them (92%) stopped attending the group sessions because they were transferred to another accommodation. The low intervention dose due to redistribution could account for the non-significant changes in PTSD and depression. It has been shown that psychological stability needs time to develop [74]. Hence participating four times in the imaginative stabilization group sessions may not be enough to evoke statistically relevant changes in symptoms. Further, culture, religion, and ethnic background might have had an influence on the results. The heterogeneity of our sample makes it difficult to make generalized assumptions concerning these factors and would need further research with a bigger sample size. Nevertheless, this longitudinal pre-post study represents an indispensable prerequisite for subsequent controlled trials.

The second limitation is that the symptom changes described in this study may also have been caused by unspecific treatment factors or a spontaneous stabilization. Both the group setting with its constant structure and the fact that the therapists remained the same throughout the study may have provided feelings of safety and support (68). The presence of other group members may either have been an inhibiting or a stabilizing factor. The study did not address factors that occurred between the imaginative stabilization group therapy sessions as well as the participants' possible improvement due to the accompanying psychopharmacological treatment. Most of the participants took mirtazapine or amitriptyline, the main aim being to improve sleep regulation.

Third, the study consisted only of male participants and the imaginative stabilization group therapy sessions were held in English. Neither the participants nor the therapists spoke English as their first language. This means that our results might not be generalizable to other groups of refugees and in addition the results might be limited due to linguistic difficulties on both sides.

Last, the results gained through the questionnaires may have been biased by social desirability, errors of central tendency, or by the participants having a different perception of emotions or bodily symptoms.

### Conclusion

This study has highlighted the application of imaginative stabilization techniques as a promising approach to treat traumatized refugees sheltered in reception centers. The imaginative stabilization techniques proved to be a feasible and effective strategy for emotional stabilization, i.e., strengthening individual resources, empowering the self, and reducing symptoms associated with distress and anxiety disorders. The results of this pilot study support the use of imaginative stabilization techniques by participants from different cultures or ethnical backgrounds, an aspect which could be investigated in more detail in future research projects. In light of the methodological limitations described above, this study should be followed up by a randomized, controlled study to confirm our results. Furthermore, it would be beneficial if imaginative stabilization techniques in other languages were included.

## Author contributions

CZ, CK, and CN conceived the study. CZ, CK, IT, DK, FJ, SZ, SH, WH, and CN participated in the design of the study. CZ, CK, and IT carried out the study. CN supervised the project. CZ and CN carried out the quantitative and qualitative analysis and finally drafted the manuscript. All authors read and approved the final manuscript.

### Conflict of interest statement

The authors declare that the research was conducted in the absence of any commercial or financial relationships that could be construed as a potential conflict of interest.
